# The impact of digital transformation on the total factor productivity of heavily polluting enterprises

**DOI:** 10.1038/s41598-023-33553-w

**Published:** 2023-04-19

**Authors:** Jinqi Su, Yinying Wei, Shubin Wang, Qilei Liu

**Affiliations:** School of Economics and Management, Xi’an University of Posts & Telecommunications, Xi’an, 710061 Shaanxi China

**Keywords:** Socioeconomic scenarios, Sustainability

## Abstract

In the digital economy, the relationship between digital transformation and a company's total factor productivity has profound implications for high-quality business development. Heavy polluters are given more environmental responsibility because of their high pollution and emission characteristics. This paper analyses the theoretical framework for the impact of digital transformation on the total factor productivity of heavily polluting firms. Using a sample of Shanghai and Shenzhen A-share heavy polluters from 2010 to 2020, we explore how the digital transformation of heavy polluters affects the total factor productivity of firms. The study found that the digital transformation of heavily polluting companies can effectively improve total factor productivity, internally by increasing their level of green technology innovation and externally by increasing their willingness and capacity for corporate social responsibility. At the same time, digital transformation can improve total factor productivity by reducing cost stickiness, revealing the "black box" in which digital transformation affects the total factor productivity of an enterprise. It was further found that the digital transformation of companies with high levels of environmental investment, large enterprises, those in non-manufacturing industries, and heavy polluters of a state-owned nature had a more significant impact on total factor productivity. The findings of the study provide empirical evidence for the digital transformation of heavily polluting companies to improve productivity and the green transformation of the economy for companies under the low carbon goal.

## Introduction

The Chinese economy has now shifted from a stage of high growth to a new stage of high quality and sustainable development. As the world's largest developing country, China's economy has experienced rapid growth over the past few decades, with heavy polluting industries, represented by electricity and steel, being the pillar industries driving economic development. 2021 The State Council's "Guiding Opinions on Accelerating the Establishment of a Sound Economic System for Green, Low-Carbon and Circular Development" states that, "To build an economic system of green, low-carbon and circular development, to improve energy use efficiency and reduce energy and resource consumption for key industries by 2030", "To adhere to the innovation drive and enhance the technological innovation capacity of enterprises", "To promote green economic transformation as well as industrial optimisation and upgrading, reduce pollution emissions and continuously improve environmental quality" and other objectives. President Xi has also pledged that China will strive to achieve carbon peaking by 2030 and carbon neutrality by 2060, hereinafter referred to as the "double carbon" target, which is a major strategic decision made by China based on its responsibility to promote the building of a community of human destiny and the inherent requirement to achieve sustainable development. As a major player in China's industrial economy and a major producer of environmental pollutants, the heavily polluting industries are more obviously affected by environmental changes and policy constraints due to their "high energy consumption" and "high pollution" characteristics and production patterns, and it can be seen that heavily polluting enterprises are facing two major challenges: industrial upgrading and low carbon transformation. In the process of their own development, the daily business activities of heavily polluting enterprises are particularly inseparable from the environment and resources, and at the same time they are the main force in promoting social construction and ecological protection of the environment, and in the pursuit of maximising economic benefits, they have to break through the bottleneck of development and reach new growth points. At the same time, we must actively assume social responsibility, implement the concept of development and environmental protection, and focus on the unity and coordination of economic, social and environmental benefits, a series that requires companies to have both stable and efficient productivity. However, the production activities of heavy polluters are accompanied by increasing inter-company differences, such as digital transformation and the level of technological innovation, which make the implementation of policies for green transformation much less effective. In the new industrial revolution with the digital economy at its core, digital technology is becoming a key to the transformation of enterprises. The widespread use of digital technology can reduce the demand for emission-intensive products, optimize resource management through system integration and help improve the environment, which is an important step towards achieving the goal of "double carbon". Since 2020, the "Special Action Plan for Digital Empowerment of SMEs" and the "Notice on Accelerating the Digital Transformation of State-owned Enterprises" have been released to help the digital transformation of enterprises. In the face of the challenges posed to heavy polluters by the "double carbon" target, the "spell" of industry being inseparable from pollution can be broken through digital transformation, driven by changes in practice and policy, which has become a new driving force for enterprises to improve productivity. At the micro level, total factor productivity fully reflects the distribution of factors and examines the relationship between the economic system, technological progress, innovation and a range of other factors and the country's economic growth. It is a comprehensive and forward-looking indicator of the efficiency of a company's output, reflecting its core competitiveness and sustainable development capacity. Therefore, exploring how the digital transformation of heavy polluters in the digital economy can improve the total factor productivity growth of companies in heavy polluting industries? is key to driving companies to become more competitive, while being able to inject new momentum into economic growth.

The White Paper on the Development of China's Digital Economy (2022) states that in 2021, the digitalization of China's industries will reach RMB 37.2 trillion, accounting for 32.5% of GDP. The integration of digital technology, the core driver of the digital economy, with the real economy not only enhances the digitalisation, networking and intelligence of enterprises, achieving efficiency improvements and structural optimisation, but also accelerates the reconfiguration of economic development and governance models. Enterprises use digital technology to provide new channels for driving digital transformation to increase productivity. There are two important influences on the total factor productivity improvement of micro enterprises, internally, there are management practices, IT and R&D, digitalization, commodity networks, innovation, etc.; External factors are market, industry and local conditions, such as the degree of competition, technological development, agglomeration economies and specialization^1^. However, the implementation of digital transformation itself is the process of using emerging information technology to reform production, operation and service methods to enhance innovation, break time and space constraints, rapidly access knowledge, promote the integration of two industries or reduce costs to improve productivity^2^. More and more companies are making digital changes with the help of emerging digital technologies. Heavy polluters are using digital means to improve their energy consumption structure, improve their processes, reduce their pollution emissions and realise intelligent changes in their production models, enabling them to transform to low carbon and thus improve their competitiveness and sustainable development. It is thus important to study the impact of digital transformation of heavy polluters on the total factor productivity of enterprises. So, can digital transformation become a new thrust for the high-quality development of heavy polluters? Does the digital transformation of enterprises help to improve the level of green technology innovation and corporate social responsibility capabilities, curb cost stickiness of enterprises and thus benefit them and ultimately improve total factor productivity?

The available literature on the digital transformation of enterprises and its economic consequences has focused on two main areas: (1) At the micro enterprise level, first, digital transformation can promote total factor productivity through mechanisms that promote the upgrading of the enterprise's human capital structure, improve technological innovation capabilities, increase capacity utilization, promote the integration of the two industries, and enhance the overall operational efficiency of the enterprise^2^, second, since knowledge is one of the most important resources among heterogeneous resources, digital transformation can enhance enterprise total factor productivity by accelerating knowledge spillover among enterprises. Digital transformation can significantly improve the information processing capability of enterprises and facilitate the flow and sharing of information and knowledge elements within the enterprise^3^, Third, digital transformation of enterprises can reduce information asymmetry, enhance positive market expectations and improve stock liquidity levels^4^. (2) At the macro effect level, green innovation in manufacturing firms in the context of digital technology application can significantly improve the current market performance of firms. It has been argued that the development of digital economy can effectively promote the development of green technology innovation and urban carbon reduction in cities^5^, enhancing entrepreneurial activity to empower high-quality development^6^. A review of the literature reveals that, although scholars at home and abroad have achieved some drawable results in their research on the economic effects of digital transformation. However, a mature system of theoretical and analytical frameworks has yet to be developed.

It can be seen that there is room for further depth in the existing literature on the impact of digital transformation on the total factor productivity of enterprises. In order to gain a clear understanding of the specific processes by which digital transformation supports the total factor productivity of enterprises, an in-depth theoretical exploration of the underlying logic and the different potential mechanisms is required. According to the existing literature, first of all, there is little literature that analyses the importance of digital transformation in terms of total factor productivity, especially for heavily polluting enterprises where productivity and environmental pollution are directly linked. Secondly, the level of green technology innovation has not been examined internally and the institutional pathway to stakeholder-based CSR has not been examined externally; Further, the existence of cost stickiness may reduce the operating performance of the business in the current period, many scholars have attributed cost stickiness to changes in the external environment and internal interest orientation of firms, but have not integrated digitalisation, cost stickiness and total factor productivity into a unified framework to analyse the impact of cost stickiness on business performance. Therefore, this study takes heavily polluting enterprises as a sample and selects heavily polluting A-share listed enterprises in Shanghai and Shenzhen from 2010 to 2020 to explore the impact of digital transformation on enterprise total factor productivity and the mechanisms of green technological innovation, corporate social responsibility and cost stickiness, and to dig deeper into the heterogeneous characteristics of digital transformation affecting enterprise behaviour.

The possible marginal contributions of this paper are mainly in the form of: ① Based on the microscopic perspective of the enterprise, and from the traditional "techno-economic paradigm" theory, revealing the path of digital transformation in the "techno-economic paradigm" of the digital economy in the era of digital economy to improve the productivity of traditional heavy polluters, the mechanisms by which digital transformation affects the total factor productivity of firms are explored and tested from a wider range of dimensions. It clearly shows that digital transformation is based on enterprise green technology innovation to improve enterprise total factor productivity, on the one hand; On the other hand, based on the alignment of external interests with stakeholders, i.e. taking on corporate social responsibility and improving the total factor productivity of the company. Finally, the introduction of cost stickiness variables reveals the "black box" of digital transformation affecting the total factor productivity of enterprises, providing a new perspective for exploring the total factor productivity of enterprises in the digital era. ② Currently, due to the lack of authoritative measurement methods for digital transformation, existing literature generally uses textual analysis to measure the overall situation of digital transformation in enterprises. (a) This study provides two measures of digital transformation, the text analytic approach and digital investment; ③ Unlike the existing studies, this paper focuses on listed companies in heavy polluting industries in the context of "double carbon" target, and empirically verifies the impact of digital transformation on total factor productivity of heavy polluting enterprises, which has important social and economic significance.

## Theoretical analysis and research hypothesis

### Digital transformation and enterprise total factor productivity

The digital transformation of enterprises can be seen as a "techno-economic paradigm" revolution in the digital economy triggered by the integration of digital technologies with the real economy, which has a significant impact on the improvement of total factor productivity of enterprises^7^. The "techno-economic paradigm" structure of the digital economy is a new leading technological system with digital technology at its core, an industrial system with digitalisation and digital industrialisation at its core and a system of social applications for the digital economy derived from the traditional "techno-economic paradigm" structure^8^. Emerging digital technologies, with big data, cloud computing and artificial intelligence at their core, are integrating deeply with the real economy, bringing about disruptive changes. In turn, the economic form that emerges first mainly causes a chain of changes in all areas of society, culminating in a techno-economic paradigm shift in the entire economy. The digital economy is essentially a new paradigm shift in the technology economy, as can be seen when the current economic structure and shape changes are contrasted with the traditional economic structure and traditional economic shape. And digital transformation facilitates the development of the digital economy, which also has a significant impact on the total factor productivity of companies. Specifically, the impact of digital transformation on the total factor productivity of enterprises is manifested in four dimensions:

Firstly, the business operation mode is optimized. On the one hand, digital transformation of enterprises can promote business model innovation^9^, restructuring traditional business, management, service and business models^10^. Digital transformation touches various aspects of operations, management, marketing and cost control, and its essential feature is to trigger a change in business model, making the reformed business model more suitable for the development of the digital era and the multi-dimensional needs of customers^11^. For example, Goldwind Technology has built a business model for industrial products services based on the integrated, platform-based and quantitative features of digital technology^12^. Red Leader Group uses a typical C2M business model, which enables customers to submit personalized requirements and participate in design using Internet technology, and this information is uploaded to the customization platform to form a digital model, and through a series of links, customization services are completed^13^, this is conducive to absorbing new production factors, adjusting and optimizing production methods, and thus improving the total factor productivity of enterprises. On the other hand, companies can optimize their production models through digital transformation: by addressing challenges such as low value-added products and multiple market needs that cannot be effectively met, use the industrial Internet platform to improve user participation throughout the product life cycle, accurately locate and analyze user needs, and implement modular and personalized design, flexible manufacturing, and intelligent warehousing based on data integration and analysis, model library sharing, and supplier collaboration to achieve a high-efficiency, zero inventory production model^14^.

Secondly, reduce corporate agency costs. Agency cost theory suggests that out of inconsistent interests and inconsistent information, agents may act to undermine the contract to the detriment of the principal. Digital governance, as an extension of digital transformation, has become a key initiative to empower internal and external oversight mechanisms and break down information asymmetries between management and governance. This is evidenced by the fact that digital transformation can lead to changes in both external transaction costs and internal control costs, thus affecting the expansion and contraction of corporate boundaries^15^: on the one hand, the improvement of enterprise digitalization can reduce internal control costs and make the management and operation of the organization more efficient, specifically, information technology under digitalization can empower organizational management, facilitate timely communication between enterprises, reduce the coordination costs of various departments, and reduce information asymmetry letters^16^, at the same time, it can realize the real-time and transparent internal control link, reduce the space for divisional speculation, and reduce the supervision cost of vertically integrated enterprises and the efficiency loss caused by divisional agency problems^17^. On the other hand, the degree of digitization can reduce the external transaction costs of enterprises. Digital technology enables timely inter-company communication and real-time tracking of materials, which ensures that the details of transactions between companies and counterparties can be flexibly adapted to immediate needs even in the case of uncertain contracts, thus reducing the production-related costs of incomplete contracts^15^. In addition, highly transparent information on creditworthiness, performance record, technology level, product quality, reputation, etc. under digital technology increases the transparency of corporate information and avoids high production-related costs due to counterparty default or potential opportunistic behavior, thus reducing external transaction costs for companies^18^. Digital technologies such as cloud computing, big data and artificial intelligence provide connectivity to enable better communication within and with the outside of the enterprise, thereby reducing transaction costs and increasing productivity.

Thirdly, to improve the innovation capacity of enterprises. A corporate innovation ecosystem is a community with a comprehensive collaborative innovation support system, in which each innovation agent, through its own heterogeneity, collaborates with other agents to create value and forms a network of interdependence and symbiotic evolution^7^. On the one hand, companies are breaking down the spatial limitations of innovation activities through digital transformation. Digital technology can integrate the internal and external resources of an enterprise and can facilitate the rapid sharing and flow of knowledge and data elements between the business systems of an enterprise's innovation ecosystem^3^, enhancing the internal control operations of an enterprise through information optimisation effects, synergy spillover effects and signal demonstration effects^19^. On the other hand, digital transformation has also changed the innovation model and innovation approach changes in the corporate innovation ecosystem. From the perspective of innovation models, digitalization encompasses many aspects of technology discovery and value discovery, as well as iterative innovation by firms through generational development, From an innovation approach perspective, the data element makes it possible for the supply and demand side to innovate in business, the supply side influences production organization, resource allocation and supply mode, enabling the intelligent transformation of production technology, while the demand side focuses on user value, promoting convenient transactions and vivid experiences, thus adjusting business models and improving overall production efficiency^20^.

Fourth, to help upgrade the industrial structure. The optimisation and upgrading of industrial structure mainly refers to the improvement of industrial efficiency and rationalisation of industrial structure, which can be divided into rationalisation of industrial structure and advanced industrial structure. According to the new structural economics theory, industrial restructuring is the process of concentrating resources within an industry towards new products or areas of high efficiency and shifting resources to high value-added industries. The digital economy provides a new economic and technological paradigm for the construction of a modern industrial system by reengineering the industrial system through the "renewal" mechanism of the digitalisation of industry acting on traditional industries. Specifically, digital innovation technology realises the rationalisation of industrial structures: the deep integration of the digital economy and the real economy, the integration of the whole sector, the whole production process, the whole factor and the industrial life cycle, and the flow of technology, logistics, capital and talent to facilitate the transfer of production factors from inefficient industries to efficient industries^21^. The flow and sharing of factors between industries has brought about changes in production methods and reshaped new models of industrial development. Digital innovation technologies for organisational change and advanced industrial structures: on the one hand, the innovative combination of digital production factors, considering data as a new production factor, promotes the digitally driven re-engineering of enterprises' production processes, procedures, supply chains, internal management and market activities, thereby improving the total factor productivity of enterprises and industries and promoting the "renewal" of industries. On the other hand, the digital transformation of production processes and the realisation of multi-system interaction in production promote flexible production organisation and process re-engineering, and smart manufacturing improves the efficiency of collaboration between systems^22^. Accordingly, the hypothesis is formulated that:

#### H1

Digital transformation has a significant positive impact on total factor productivity of enterprises.

Through the above analysis, digital transformation promotes the total factor productivity of enterprises by optimising their operation mode, reducing their agency costs, improving their innovation capability and helping to optimise and upgrade their industrial structure. However, there may be other pathways for the impact of digital transformation on the total factor productivity of heavily polluting firms when the focus of our study is on them. Therefore, this paper explores the impact of digital transformation on the total factor productivity of enterprises in terms of the level of green technology innovation, corporate social responsibility and cost stickiness paths.

### Digital transformation, green technology innovation and total factor productivity of enterprises

Green technology innovation is a new type of technology innovation that integrates green design, development, production and marketing concepts into the whole life cycle of products in the innovation process, so as to achieve green and refined management in all stages of the enterprise life cycle and realize economic-ecological-social benefits^23^. In the wave of digitalization enterprises through digital transformation can largely empower the level of green technology innovation. Optimize and reorganize product design, R&D process, resource utilization, etc. So that enterprises can abolish high energy-consuming, high-polluting and low-efficiency production technologies, effectively control pollution emissions and resource waste, accelerate the elimination of backward production capacity, and instead achieve energy saving and emission reduction through green technology innovation^28^. Achieve the effect of win–win economic-ecological-social benefits, and improve enterprise production efficiency and management efficiency.

Digital transformation provides new ideas to improve the level of green technology innovation and total factor productivity of enterprises. According to the theory of natural resource-based view, the internal organizational elements such as technological capability of enterprises are the key to their green technological innovation and to improve their core competitiveness. In the era of digital economy, the integration of resources needs to be realized and accomplished with the help of next-generation information and communication technologies. First, digital technology can promptly identify enterprise value demands and effectively solve them, and its information detection function allocates funds and other qualitative resources to "green technology innovation projects" carried out by enterprises with outstanding economic benefits, rational use of resources, and coordinated development of environment and economy, forcing the "three high" enterprises to carry out green technology transformation, At the same time, the environmental data is fed back to the environmental governance department, and the environmental governance achieves an efficient closed-loop^24^, which promotes the development of green innovation in manufacturing enterprises. Second, enterprise digitization can promote information sharing and knowledge integration, and help optimize innovation technology resources. On the one hand, enterprise digitalization can produce information sharing effect to accelerate the integration of internal and external resources of the enterprise, to obtain, share and reorganize resources by enhancing communication sharing and information interchange of the enterprise, to explore the existing resources and potential innovation value, to provide resource base for green innovation, to create high compound value of resources, so as to optimize the innovation technology resources of the enterprise^25^. On the other hand, from the level of knowledge integration effect, green technology innovation integrates knowledge information from different fields, such as enterprise production, pollution reduction and energy consumption reduction, its green innovation process should create, integrate and diffuse knowledge from different fields within the organization^26,27^, and rationalize the use of internal and external knowledge in order to deeply understand the key technologies of green technology innovation. In contrast, enterprise digitization promotes collaborative and open innovation^28^, which helps companies to integrate and reconfigure knowledge elements from different technological domains and stimulates them to engage in green innovation. Third, based on environmental adaptation theory, the rapid changes in the external environment require companies to react and integrate quickly to adapt to the changes in the environment, while the use of digital technology can promote companies to adapt to external environmental shocks and get rid of organizational practices, providing new impetus for companies to implement green innovation technologies and expand the green innovation boundary. Fourth, the digital economy has improved the efficiency of resource allocation and real-time monitoring of the ecological environment, effectively reducing pollutant emissions; at the same time, the integration and development of the digital economy and the real economy have promoted innovation output, enhanced the efficiency of green innovation and reduced innovation costs. Especially for heavy polluting enterprises, the realization of "energy saving and emission reduction" needs the strong support of technological innovation, so heavy polluting enterprises need to focus on improving green technology innovation capabilities.

The improvement in the level of green technology innovation further enhances the total factor productivity of enterprises. First of all, green innovation is a new type of innovation that combines the advantages of both environmental protection and economic development. The effective play of green innovation as a green technology can confer competitive advantages to enterprises through isolation mechanisms and technology spillover^29^, and the application of green technology innovation can reduce costs, achieve green product differentiation, and other advantages, which can better compensate for the initial investment while bringing more benefits to enterprises and enhance the growth advantages of stakeholders by improving their environmental management systems and ultimately promote productivity^30^. Second, corporate green technology innovation has a natural advantage in the economic theory of corporate profit maximization. Green innovation activities by companies, whether it is green product innovation or process innovation, can increase the productivity of industry. Green product innovation can generate innovation spillover to upstream and downstream industries through the backward and forward linkage effect, and upstream and downstream supporting industries will strive to improve their own technology in order to meet the technological requirements of new product development enterprises, which will lead to a gradual increase in overall TFP. Green process innovation enables enterprises to reduce production costs per unit of output or increase output per unit of time while ensuring product quality, thus enabling them to optimise production processes, reduce the level of consumption of factor resources, increase output per unit of factor and promote efficiency change^31^. Furthermore, green technology innovation by enterprises to produce green products, or green technology innovation to reduce energy consumption and pollution emissions, quickly improve the negative image of heavy pollution enterprises in the public "high pollution, high emissions", improve the production technology, improve the unit output, effectively expand the market share of products, and improve the efficiency of enterprise production. In addition, under the change of national policy direction, the government strengthens the rigid constraints on enterprises to save energy and reduce consumption, pollution and carbon emissions, and focuses on promoting low-carbon development, green development, circular development, adjusting and optimizing industrial structure. Environmental subsidies from the government and financial institutions to some extent support the shortage of funds generated by the environmental investment behavior of enterprises in green technology innovation activities, improving their financing capacity and reducing environmental risks. Finally, after green technology innovation, firms can also transfer the green innovation technology and receive patent technology transfer fees, which contributes to the total factor productivity of the firm^32^. Accordingly, it is hypothesized that:

#### H2

Digital transformation improves enterprise total factor productivity by promoting green technology innovation levels.

### Digital transformation, corporate social responsibility and corporate total factor productivity

Corporate social responsibility mainly refers to the responsibility of companies to their employees, consumers, suppliers, communities, non-profit organizations and the environment, while generating profits and taking corresponding social responsibility to shareholders^33^. The fulfillment of corporate social responsibility includes the identification of social and environmental issues, the selection of social responsibility strategies and issues, and the participation of stakeholders in value creation. As a "high pollution" and "high emission" enterprise, heavy polluting enterprises have to bear the comprehensive responsibility of social, economic and environmental aspects. The Chinese government has also introduced a series of policies and regulations, including the new PRC Environmental Protection Law, and began issuing social responsibility standards at the national level in 2016. Under the dual pressure of social opinion and government control, heavily polluting enterprises have been paying more attention to social responsibility and have continued to integrate social responsibility concepts into their practices.

The digital transformation of enterprises is based on digital technology, which enables value co-creation for all stakeholders and brings new impetus to the fulfilment of corporate social responsibility. On the one hand, digital transformation enhances the willingness of enterprises to fulfill their social responsibility: The unique openness, co-creation and sharing nature of digital technology enables different interests to participate in the enterprise decision-making process^34^, Based on stakeholder theory, digital transformation innovates corporate business models, forming a network chain that integrates multiple groups such as shareholders, suppliers and consumers. In order to attract more quality resources and maintain a good social image, companies will actively engage in social responsibility to attract more stakeholders to join them, thus obtaining the expression of stakeholders' demands. On the other hand, digital transformation improves the ability of companies to fulfill their social responsibility: Companies using digital technology can quickly identify social and public environmental issues. For example, intelligent algorithms and blockchain technology are used to build optimal models and find the best solutions. By building or supporting the formation of a social resource integration platform, different social and environmental pain points are captured and identified in order to analyze the value propositions of multiple stakeholders, match the company's superior resources with them, and finally implement them into specific social responsibility issues to build a link between the company and society. Help strategic decision makers analyze, identify and select CSR issues from a more objective and open perspective, and promote the fulfillment and quality improvement of social responsibility. For example, by launching a social "energy conservation and emission reduction coalition" to leverage resources from relevant enterprises, NGOs and even governments or directly implement climate change issues^35,36^.

The active implementation of CSR can directly contribute to the improvement of total factor productivity. First, heavy polluters have an obligation to fulfill their social responsibility for environmental protection. According to signaling theory and social reputation effect, on the one hand, heavy polluters' concern for social and environmental responsibility can demonstrate to society the value contribution they make, as well as to stakeholders the good reputation and sustainable management philosophy of the enterprise, attracting more investors' attention, enabling the expansion of production scale and eventually increasing enterprise productivity. On the one hand, heavy polluters' concern for social and environmental responsibility can demonstrate to society the value contribution they make, as well as to stakeholders the good reputation and sustainable management philosophy of the enterprise, attracting more investors' attention, enabling the expansion of production scale and eventually increasing enterprise productivity^37^. Second, according to resource dependency theory, due to the uncertainty of the environment and the lack of sufficient resources, heavily polluting enterprises rely on the pursuit of more resources from the outside to reduce the adverse impact due to the external environment; Moreover, the daily business activities of a company need to coordinate with its stakeholders to help the company obtain potential resources and promote the sustainability of business operations. And companies actively fulfilling their positive corporate social responsibility can receive more quality resources for production and shareholder support from government and other institutions encouraging their R&D and innovation, etc. By promoting environmental technology innovation and production technology innovation through synergy with government and universities, integrating information, and using new resources and capabilities formed by the application of high-tech and promotional technologies to improve pollutant treatment processes, optimize production processes, and reform management systems, total factor productivity is gradually rising in stability^33^. Accordingly, the hypothesis is formulated that:

#### H3

Digital transformation improves total factor productivity by increasing the willingness and ability of companies to fulfill their social responsibility.

### Digital transformation, cost stickiness and enterprise total factor productivity

Cost stickiness is mainly reflected in the asymmetry between cost and business volume, which is more significant when business volume increases than when business volume decreases, especially when the high cost stickiness caused by resource redundancy and resource mismatch reduces the efficiency of resource allocation^38^. The integration of the digital economy with the real economy is seen as a key measure to achieve effective cost management and optimal resource allocation^39^. Digital transformation can replace and innovate traditional manufacturing methods and curb the cost stickiness of companies from three perspectives: agency costs, adjustment costs and management's optimistic expectations.

The first is analysed in terms of agency costs. Digital transformation can mitigate agency risk arising from information asymmetry letters and curb cost stickiness. The use of digital technology makes it easier for principals and agents to communicate with each other by increasing the transparency and speed of information transmission, thus reducing the moral hazard of agents arising from information asymmetry, motivating agents to work towards the goal of maximising the interests of the principal and alleviating the problem of high cost stickiness^40^. In addition, with a digital management model based on collaborative sharing, dynamic supervision and big data communication, it is easier for principals to form effective monitoring of agents' behaviour, avoiding moral hazard due to information asymmetry, reducing cost stickiness and promoting the improvement of enterprise productivity. Secondly, an analysis in terms of adjustment costs. Digital transformation can reduce asset realignment costs, thereby curbing cost stickiness. In the traditional model, there are high adjustment costs for business assets and it is difficult to convert them to other uses^38^. Digital transformation can facilitate the cross-border integration of businesses into the digital economy and alleviate problems such as adjustment costs constraining corporate R&D activities. Under the digital platform model, companies have relatively lower resource alignment costs and consequently lower cost stickiness, which in turn leads to improved performance levels. When business volumes rise, the low-cost resource space of the digital platform provides sufficient information to help managers accurately forecast the resources needed to expand production capacity, meet the operational needs of expanded production and achieve productivity gains. When business volumes drop, management can identify idle and redundant resources that can be cut based on the Collaboration Cloud platform, improving resource allocation rates and reducing adjustment costs, thus reducing cost stickiness^41^. The reduction in adjustment costs helps the company to adjust its assets in a timely manner and improve the efficiency of asset utilization, which promotes the reduction of the company's cost stickiness and brings about an increase in the total factor productivity of the company^38^. Thirdly, analysis from the perspective of managers' optimistic expectations. Digital transformation dampened management's optimistic expectations and suppressed cost stickiness. By automating business processes, digital transformation can reduce human intervention and reduce the scope for self-interested manipulation by management, which can curb the cost stickiness of the company. On the one hand, companies can use digital technology platforms to build more complete databases of customer consumption information and accurate sales forecasting models, enabling managers to forecast future market demand more accurately, correcting optimistic forecasting biases caused by management's overestimation of future market demand and thus reducing cost stickiness. On the other hand, enterprises achieve intelligent management and effective cost control by means of digital technology such as big data, artificial intelligence and cloud computing^42^. In summary, the digital transformation of enterprises can reduce the cost stickiness of enterprises, make enterprise resources flexible and change according to market changes, improve the efficiency of resource allocation, expand the profit margin of enterprises, and thus improve the total factor productivity of enterprises. Accordingly, the hypothesis is formulated that:

H4: Digital transformation improves total factor productivity by reducing the cost stickiness of traditional businesses.

## Study design

### Sample selection and data sources

This paper selects the heavy pollution enterprises listed in Shanghai and Shenzhen A-shares in China's heavy pollution industry from 2010 to 2020 as the research sample. Heavy pollution industry is the target of supply-side structural reform, that is, de-capacity, de-inventory, de-leverage, cost reduction and short-board, which is the key monitoring object of China environmental protection department. It is of great significance and practical significance to study the impact of digital transformation on the total factor productivity of heavy pollution enterprises. According to the "Guidelines for Disclosure of Environmental Information of Listed Companies" issued by the Ministry of Environment of China in 2010 as the assessment criteria for heavily polluting industries, including 16 categories of industries such as thermal power, iron and steel, chemical and pharmaceutical, combined with the 2012 "Guidelines for Industry Classification of Listed Companies" issued by the China Securities Regulatory Commission. In this paper, with reference to scholars' determination of the codes of heavy pollution industries^43^, and the listed companies in Shanghai and Shenzhen A shares with industry codes B06, B07, B08, B09, B10, B11, B12, C17, C18, C19, C22, C25, C26, C27, C28, C29, C31, C32, and D44 are identified as the research objects of this paper. As the rapid development of digital technology is mainly concentrated in recent years, the sample time span selected for this paper is 2010–2020 in order to highlight the impact of digital development on heavily polluting enterprises. In this paper, the raw data were processed as follows. (1) exclude ST and ST* listed companies; (2) exclude data of companies with missing financial data. (3) To control for the effect of extreme values, tailoring was applied to all continuous variables at the 1% and 99% quartiles. The data sources for this paper are as follows: Digital transformation data from a combination of annual reports disclosed to the public by listed companies and actual applications; Green patent data source CNRDS database; CSR data from Hexun.com CSR database; All the rest of the data are obtained from the CSMAR database.

### Variable definition

#### Explanatory variables

Total factor productivity of enterprises (tfp). There are various estimation methods on total factor productivity measurement such as OLS fixed effects, FE method, OP method, LP method, and GMM method. The estimated results reflect only individual heterogeneity due to the endogeneity problem caused by the possible simultaneity bias of the OLS method. While the OP method can effectively avoid simultaneity bias and selectivity bias, the LP method takes the intermediate goods input as a proxy variable from the perspective of data, and the data selection is more flexible, which overcomes the problem of reduced estimation accuracy caused by the loss of a large number of invalid samples. The use of semi-parametric estimation (e.g., OP, LP) can effectively avoid the endogeneity problem in the above traditional estimation methods^44^ and is suitable for firm-level TFP estimation. Therefore, in this paper, we refer to the study of Lu and Lian^44^ and use the LP method for TFP estimation and the OP method for robustness testing.

#### Explanatory variables

Digital transformation level (Dig). The existing literature has three ways of measuring the degree of digital transformation of enterprises: one is through text analysis method^4^, secondly, the amount of digitization-related portion disclosed in the notes to the financial report of the listed company as a percentage of the total intangible assets^45^, Third, through questionnaires^46^, these three ways to get data to measure the level of digital transformation of the enterprise. Given the limited sample size, the lack of easy access and the low representativeness of the questionnaire approach. Based on this, this paper refers to Wu et al.^4^, Yuan et al.^15^ and others who used text mining method to obtain Dig, using python crawler function to gather and organize the annual reports of heavy polluters listed in Shanghai and Shenzhen A shares in heavy pollution industry, Through the machine learning method, we collected the word frequencies of different keywords in the "Management Discussion and Analysis" section of the annual reports of listed companies, and based on "jieba" Chinese word splitting software in Python, we divided the words of MD&A content pairs and eliminated negative words such as "no", "not", "none", etc. We analyzed the word frequencies of the data pool formed by extracting the text of annual reports of listed companies, and counted the number of word frequencies of digital keywords in MD&A of each company.

#### Intermediate variables

① Green Technology Innovation (GI). Existing research on the measurement of the level of green technology innovation has two main perspectives: input and output. In this paper, the number of independent green patent applications by listed companies is used as the core explanatory variable. Green patents can visually reflect the output of green innovation of enterprises, considering that the patent application cycle is long and has certain risks and uncertainties. And the internationally accepted green patent classification system is the "IPC Green Inventory" launched by WIPO (World Intellectual Property Organization) in September 2010. Because design patents do not adopt IPC classification, this paper refers to Xu et al.^47^ who used green patent application data (including the number of invention-type and utility model) to measure the level of green innovation output of listed companies and did logarithmization of the number of green patents applied by listed companies in the current year to eliminate the effect of quantiles. **②** Corporate Social Responsibility(CSR). Drawing on the methodology of Xiao^36^ and others, this paper uses Hexun CSR rating data to measure the social responsibility performance of companies in the current year. The index evaluates overall corporate social responsibility in five areas: shareholders, employees, suppliers and consumer rights, environment, and social responsibility. ③ Cost stickiness (JSTICKY). In this paper, WEISS is chosen to propose a metric model to measure the cost stickiness of micro enterprises: the WEISS model, which measures the degree of cost stickiness of enterprises^48^.

$$JSTICKY_{i,t} = \log (\Delta \cos t_{i,t1} /\Delta sale_{i,t1} ) - \log (\Delta \cos t_{i,t2} /\Delta sale_{i,t2} ),t_{1} ,t_{2} \in (t,t - 3)$$

where i represents the firm and t_1_ and t_2_ denote the quarters in which the sample firm's total revenue fell and rose near the end of the four quarters of the year; ΔCost represents the amount of change in total operating costs for the quarter, where total operating costs comprise operating costs and period expenses in the financial statements; ΔSale represents the amount of change in total operating income for the quarter. Since the WEISS model calculates negative values for cost stickiness, JSTICKY is taken as an absolute value in order to test the impact of the co-existence of smart transformation and cost stickiness on firm performance.

#### Control variables

Referring to the existing studies on total factor productivity of firms, the relevant variables are controlled for. In this paper, we control for firm size (Size), firm age (Age), equity concentration (Shrcr1) gearing ratio (Lev), Total Return on Assets (ROA), board size (Board), and percentage of independent directors (Independent). The calculation methods and definitions of all variables are shown in Table 1.

**Table 1:** Variable definitions.

variable type	Name	Abbreviations	Definition
Explained variables	Total factor productivity of enterprises	tfp	LP, OP method to calculate total factor productivity of enterprises
Explanatory variables	Degree of digital transformation	Dig	Logarithm of digital transformation word frequency
Intermediate variables	Green technology innovation	GI	Ln(1 + number of green patents independently applied by enterprises)
	Corporate social responsibility	CSR	Hexun CSR Database Stakeholder Composite Score
	Cost stickiness	*JSTICKY*	Using the WEISS model to measure the degree of cost stickiness of a company
Control variables	Enterprise size	Size	Natural logarithm of total assets
	Business age	Age	Ln (current year year − year of establishment + 1)
	Shareholding concentration	Shrcr1	Percentage of shareholding of the largest shareholder
	Gearing ratio	Lev	Total liabilities/total assets* 100%
	Total return on assets	Roa	Net income/[(total assets ending balance + total assets opening balance)/2] * 100%
	Board size	Board	Natural logarithm of the number of directors
	Percentage of independent directors	Independent	Number of independent directors/number of directors

### Model construction

Based on the analysis in this paper, in order to explore the impact of digital transformation on the total factor productivity of enterprises, a benchmark model1 is constructed for empirical analysis:

1 $$tfp_{i,t} = \alpha_{0} + \alpha_{1} Dig_{i,t} + \sum {\alpha_{n} } controls_{i,t} + F{\text{irm}}_{i} + year_{t} + \varepsilon_{i,t}$$

In model (1), the subscripts i and t denote firms and years, respectively. the explanatory variable: *tfp*_*i,t*_ represents the total factor productivity of the firm; the core explanatory variable: *Dig*_*i,t*_ measures the level of digital transformation of the firm; *controls*_*i,t*_ is the set of control variables at the firm level, *Firm*_*i*_: corporate fixed effects, *year*_*t*_: for year fixed effects have also been added, $\varepsilon_{it}$ is a random disturbance term, and the coefficients portray the effect of digital transformation on the total factor productivity of the firm.

## Empirical results and analysis

### Descriptive statistical analysis

Descriptive statistics of the variables are shown in Table 2.The mean value of total factor productivity (tfp) of enterprises is 9.045, with a maximum value of 12.07 and a minimum value of 5.967, indicating that the level of total factor productivity of enterprises at the overall level of the study sample is better, but there are still significant gaps; Meanwhile, the maximum value of enterprise digital transformation level (Dig) is 4.673, the minimum value is 0, and the mean value is 0.863. The difference of digital capability among enterprises is significant, which indicates that the digital level of heavy pollution enterprises in China is lagging behind and still needs to be improved. The mean value of green patent is 0.172, the maximum value is 3.332 and the minimum value is 0. This indicates that there is a certain difference in the number of green patent applications between enterprises in heavy pollution industries. The mean value of CSR is 22.32, the maximum value is 89.01, and the minimum value is negative, indicating that the performance of China's heavy polluting enterprises in fulfilling CSR is not optimistic, which is also related to the frequent occurrence of environmental pollution incidents caused by heavy polluting enterprises in recent years, such as the pollution of Haoyuan Cement and the cadmium pollution of Longjiang River in Guangxi. The mean value of firm cost stickiness is 0.715, with a maximum value of 10.29 and a minimum value of 0.

**Table 2:** Descriptive statistics.

Variable	Abbreviations	N	Mean	p50	SD	Min	Max
Total factor productivity of enterprises	tfp	2750	9.045	9.007	0.857	5.967	12.07
Degree of digital transformation	Dig	2750	0.672	0	0.863	0	4.673
Green technology innovation	GI	2750	0.172	0	0.425	0	3.332
Corporate social responsibility	CSR	2750	22.32	20.80	13.64	− 9.540	89.01
Cost stickiness	JSTICKY	2750	0.720	0.424	0.845	0	10.29
Enterprise size	Size	2750	3.093	3.088	0.053	2.948	3.282
Gearing ratio	Lev	2750	0.421	0.415	0.201	0.014	1.398
business age	Age	2750	2.864	2.890	0.440	1.386	7.611
Shareholding concentration	Shrcr1	2750	35.40	33.34	14.62	5.048	85.23
Board size	Board	2750	2.146	2.197	0.197	1.386	2.890
Percentage of independent directors	Independent	2750	0.372	0.333	0.053	0.200	0.667
Total return on assets	Roa	2750	0.045	0.040	0.062	− 0.408	0.466

### Correlation analysis

Correlation analysis was performed between the variables of interest in the model, as seen in Table 3 Pearson correlation test results for all variables. The correlation coefficient between digital transformation and total factor productivity of enterprises is significantly positive at the 1% level, indicating that the digital level capability significantly improves the total factor productivity of heavily polluting enterprises; In terms of control variables, all control variables, except for the percentage of independent directors, have a significantly positive correlation coefficient with tfp at the 1% level. The results initially validate some of the hypotheses in this paper.

**Table 3:** Correlation analysis.

Variable	tfp	Dig	GI	CSR	JSTICKY	Size	Lev	Age	Shrcr1	Board	Independent	Roa
tfp	1											
Dig	0.166***	1										
GI	0.209***	0.038**	1									
CSR	0.226***	0.060***	0.038**	1								
JSTICKY	− 0.095***	− 0.094***	− 0.066***	− 0.048**	1							
Size	0.849***	0.074***	0.128***	0.105***	0.019	1						
Lev	0.344***	− 0.054***	0.067***	− 0.177***	0.024	0.465***	1					
Age	0.092***	− 0.022	− 0.012	− 0.092***	0.024	0.170***	0.120***	1				
Shrcr1	0.241***	0.032*	0.022	0.143***	− 0.045**	0.208***	0.032*	− 0.065***	1			
Board	0.189***	− 0.097***	0.077***	0.108***	0.059***	0.254***	0.181***	0.085***	− 0.020	1		
Independent	− 0.013	0.106***	− 0.031	− 0.023	− 0.002	− 0.016	− 0.056***	− 0.017	0.068***	− 0.525***	1	
Roa	0.168***	0.063***	0.023	0.473***	− 0.074***	− 0.071***	− 0.425***	− 0.037*	0.068***	− 0.042**	0.028	1

T-statistics are shown in parentheses. ***, ** and * indicate 1%, 5% and 10% respectively.

### Testing the direct effect of digital transformation on the total factor productivity of firms

#### Baseline regression and discussion

In order to test the hypothesis 1: the impact of digital transformation on the total factor productivity of enterprises, the regression analysis of model (1) is applied to investigate the impact of digital transformation on the total factor productivity of enterprises, and the regression results are shown in columns (1)–(3) of Table 4. Column (1) considers separately the regression results of the impact of digital transformation on the total factor productivity of firms, and the results show that the regression coefficient of digital transformation is 0.1496, which is significantly positive at the 1% level. The regression coefficient for the level of digital transformation remains significant at the 1% level after adding control variables and controlling for firm and year fixed effects. The test analysis shows that the digital transformation of heavy polluting enterprises significantly improves the total factor productivity of enterprises, and hypothesis H1 is supported. As mentioned in the theoretical analysis, the digital application of heavy polluting enterprises improves the production process, realizes the "win–win" of production efficiency and energy saving and emission reduction through intelligent collaborative application and management, realizes lean management, and promotes the development of green technology innovation. This effectively promotes the transformation of heavy polluting enterprises to low-carbon, clean and green environmental protection, reduces energy consumption and pollution emissions, enhances market competitiveness and realizes high-quality development of enterprises.

**Table 4:** Impact of digitization on total factor productivity of firms.

Variable	(1)	(2)	(3)
	tfp	tfp	tfp
Dig	0.1496***	0.05310***	0.03842***
	(13.5018)	(6.9475)	(3.7616)
Size		12.527***	10.918***
		(56.8458)	(11.9706)
Lev		0.3532***	0.3417***
		(7.8446)	(3.8974)
Age		− 0.08734***	− 0.1319
		(− 3.3979)	(− 1.0366)
Shrcr1		0.001533**	0.0004051
		(2.3467)	(0.2667)
Board		0.001197	− 0.01624
		(0.0259)	(− 0.2676)
Independent		− 0.1497	− 0.1873
		(− 1.0521)	(− 1.0568)
Roa		2.8184***	2.5193***
		(29.6374)	(14.1142)
Year	Not control	Not control	Control
Firm	Not control	Not control	Control
_cons	8.8765***	− 29.777***	− 24.624***
	(281.2839)	(− 46.2078)	(− 9.0508)
N	2750	2750	2750
R^2^	0.0661	0.5951	0.6357

T-statistics are shown in parentheses. ***, ** indicate 1%, 5% respectively.

#### Endogenous problems

##### Instrumental variables method

The digital transformation of enterprises will face heavy challenges, such as large initial capital investment, low level of digital technology, uncertain results, and talent gaps. In practice, companies with high productivity and operational capabilities are more willing to promote digital transformation, and there may be an inverse causal relationship between digital transformation and total factor productivity of companies, leading to endogeneity problems in the model. In order to test whether there is an endogeneity problem in this paper in which the independent and dependent variables are mutually dependent, a two-stage instrumental variables method regression was conducted. In this paper, the one-period lagged independent variable LDig was used as the instrumental variable for the independent variable and regressed, the results are presented in column (1) of Table 5, the regression coefficient value of 0.7869 is positive and significant at the 1% level, indicating that the instrumental variable was chosen with reasonableness and objectivity. The fitted values, LDig_hat, were derived from the first stage regression and carried over to the second stage regression, with the results listed in column (2) of Table 5. It can be seen that the value of the LDig_hat regression coefficient is 0.1392 and is significant at the 1% level. The above results indicate that there is no mutual causality endogeneity between the independent and dependent variables, further suggesting that the digital transformation of heavy polluters significantly improves the total factor productivity of enterprises.

**Table 5:** Endogeneity tests: regression results of the instrumental variables method and PSM method.

Variables	LDig as a tool variable		ADig_median as a tool variable		PSM
	Stage 1	Stage 2	Stage 1	Stage 2	
	(1)	(2)	(3)	(4)	(5)
	Dig	tfp	Dig	tfp	tfp
LDig	0.7869***				
	(32.174)				
LDig_hat		0.1392***			
		(6.7561)			
ADig_median			0.6473***		
			(13.3756)		
ADig_hat				0.1449***	
				(4.5341)	
Dig					0.037***
					(3.639)
Size	0.4137	13.4511***	2.6309***	13.2765***	11.165***
	(1.3026)	(39.733)	(5.3059)	(40.186)	(12.392)
Age	− 0.0589**	− 0.102***	− 0.1677**	− 0.0758**	− 0.13
	(− 2.0023)	(− 3.3119)	(− 2.3726)	(− 2.1131)	(− 1.013)
Shrcr1	0.0014	0.0025**	0.001	0.0023**	0
	(1.5183)	(2.1671)	(0.7135)	(2.2573)	(0.136)
Lev	− 0.0289	0.261***	− 0.1429	0.3573***	0.276***
	(− 0.3094)	(2.8193)	(− 1.0752)	(4.2854)	(3.486)
Roa	0.3596	3.0582***	0.7564**	3.2551***	2.556***
	(1.2549)	(12.8689)	(2.2553)	(16.6125)	(14.359)
Board	− 0.1725*	0.076	− 0.645***	0.1405	− 0.018
	(− 1.6799)	(0.7721)	(− 3.4113)	(1.528)	(− 0.278)
Independent	0.0195	− 0.042	0.0727	− 0.0606**	− 0.146
	(0.5358)	(− 1.3036)	(1.1969)	(− 2.1167)	(− 0.789)
_cons	− 0.8517	− 32.59***	− 6.3237***	− 32.3729***	− 25.355***
	(− 0.9196)	(− 32.7977)	(− 4.2808)	(− 33.8388)	(− 9.436)
Year	Control	Control	Control	Control	Control
Firm	Control	Control	Control	Control	Control
N	1526	1526	2750	2750	2728
R^2^	0.6194	0.8082	0.2922	0.7876	0.642

T-statistics are shown in parentheses. ***, ** and * indicate 1%, 5% and 10% respectively.

This paper generates the year-segment median of digital transformation indicators (ADig_median) through stata as an instrumental variable for the core independent variables. The instrumental variables were regressed on the independent variables and the results are presented in column (3) of Table 5, at which point the regression coefficient value of 0.6473 is significant at the 1% level, indicating that the instrumental variables were chosen with reasonableness and objectivity. The fitted value ADig_hat was derived from the first stage regression and carried over to the second stage for regression, the results of which are listed in column (4) of Table 5. It can be seen that the ADig_hat regression coefficient value is 0.1449 and is significant at the 1% level. The above results further illustrate the basic findings of this paper and that there is no endogeneity problem in this paper where the independent and dependent variables are mutually causal.

##### Propensity score matching method

**Figure 1:**
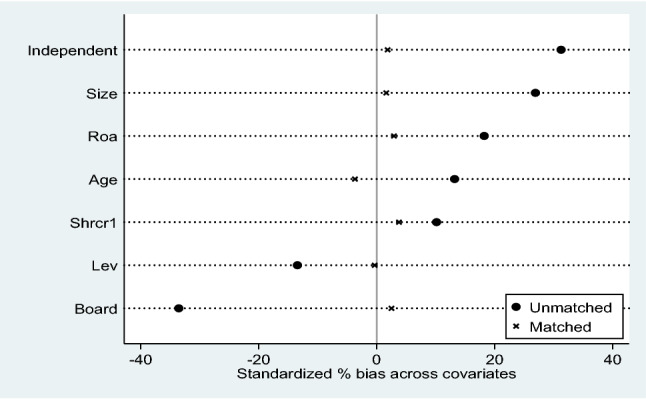
Propensity score matching test.

The PSM (Propensity Score Matching Method) method is used to test for endogeneity problems that may be caused by sample self-selection. The full sample was grouped according to whether or not the firm underwent digital transformation, and control variables consistent with model (1) were selected to match the samples in the experimental and control groups. The radius matching principle was used to find a control group with similar characteristics for the treatment group, and the radius matching results obtained are shown in Fig. 1. It can be concluded that the individual differences in the control variables between the experimental and control groups were significantly reduced before and after matching. The regressions were performed after the propensity score matching was completed and the post-matching estimates shown in column (5) of Table 5 remain consistent with the regression results of the hypothesis 1 test section. It is shown that the study findings remain robust after overcoming the problem of sample self-selection bias, indicating that the digital transformation of heavy polluters promotes the total factor productivity of enterprises.

#### Robustness tests

##### Replace the interpreted variable

In the benchmark regression analysis, the LP method is used to measure the total factor productivity of enterprises. Since different variable measures have a non-negligible impact on the conclusions of the experiment, the OP method is used to measure the total factor productivity of enterprises (top) again to do the robustness analysis. The results in columns (1)–(3) of Table 6 show that the positive contribution of digital transformation to firms' total factor productivity remains significant and the regression results are consistent with the above.

**Table 6:** Replace the regression result of the explained variable.

Variables	(1)	(2)	(3)
	top (total factor productivity)	top (total factor productivity)	top (total factor productivity)
Dig	0.1192***	0.04237***	0.03610***
	(12.0013)	(5.2177)	(3.4031)
Size		8.3605***	6.7739***
		(35.5350)	(8.9743)
Lev		0.1848***	0.1967*
		(3.8603)	(1.9411)
Age		0.02141	− 0.2403*
		(0.7799)	(− 1.6546)
Shrcr1		0.0007152	0.001073
		(1.0271)	(0.6882)
Board		− 0.03695	0.01613
		(− 0.7515)	(0.2336)
Independent		− 0.2368	− 0.2545
		(− 1.5673)	(− 1.2930)
Roa		2.5934***	2.3098***
		(25.7024)	(11.5031)
Year	Not control	Not control	Control
Firm	Not control	Not control	Control
_cons	6.5308***	− 19.355***	− 13.996***
	(263.0051)	(− 28.1331)	(− 5.9093)
N	2750	2750	2750
R^2^	0.0548	0.4353	0.5047

T-statistics are shown in parentheses. ***, * indicate 1%, 10% respectively.

##### Substitution explanatory variable

This paper replaces the core explanatory variables to verify the empirical results. Drawing on scholarly research^49^, the adoption of digital technologies needs to be based on digital investment. In order to analyse the different impacts that different digital technologies may have on a firm's total factor productivity, digital transformation is measured in terms of digital investment. Digital investment is divided into investment in hardware facilities and investment in software facilities, where hardware investment is expressed as the ratio of the net value of fixed assets in terms of computers, communication equipment and electronic equipment to total net assets, and software investment is expressed as the ratio of the net value of software assets in terms of intangible assets to total net assets. The regression results in Table 7 (1)–(6) show that both investment in hardware and software facilities can contribute to the improvement of total factor productivity, and the promotion effect of hardware investment is more obvious, indicating that there is still much room for improvement in software investment.

**Table 7:** Regression results with replacement of explanatory variables.

Variables	Software			Hardware		
	(1)	(2)	(3)	(4)	(5)	(6)
	tfp	tfp	tfp	tfp	tfp	tfp
Software	32.784***	15.523***	11.105***			
	(5.9715)	(4.4856)	(3.3536)			
Hardware				11.360***	12.385***	6.7828**
				(3.3258)	(5.9601)	(2.3227)
Size		12.687***	10.953***		12.711***	10.979***
		(57.5074)	(12.0069)		(57.9990)	(12.1711)
Lev		0.3342***	0.3409***		0.3204***	0.3318***
		(7.4185)	(3.8859)		(7.1383)	(3.7898)
Age		− 0.05604**	− 0.1184		− 0.04260*	− 0.08858
		(− 2.1933)	(− 0.9282)		(− 1.6776)	(− 0.6862)
Shrcr1		0.001543**	0.0005928		0.001130*	0.0001915
		(2.3370)	(0.3864)		(1.7172)	(0.1256)
Board		− 0.003432	− 0.008416		− 0.02531	− 0.01986
		(− 0.0740)	(− 0.1355)		(− 0.5474)	(− 0.3193)
Independent		− 0.09576	− 0.1455		− 0.1411	− 0.1836
		(− 0.6725)	(− 0.8065)		(− 0.9923)	(− 1.0205)
Roa		2.7856***	2.5028***		2.8111***	2.5184***
		(29.3411)	(14.1308)		(29.6183)	(14.0359)
Year	Not control	Not control	Control	Not control	Not control	Control
Firm	Not control	Not control	Control	Not control	Not control	Control
_cons	8.9471***	− 30.341***	− 24.800***	8.9576***	− 30.379***	− 24.904***
	(278.1861)	(− 47.1657)	(− 9.0923)	(279.5396)	(− 47.5128)	(− 9.2465)
N	2750	2750	2750	2750	2750	2750
R^2^	0.0187	0.5946	0.6333	0.0017	0.5942	0.6332

T-statistics are shown in parentheses. ***, ** and * indicate 1%, 5% and 10% respectively.

### Heterogeneity analysis

Taking into account the facts about the characteristics of the digital transformation of Chinese listed companies, this paper further analyses the results from the perspective of the level of investment in environmental protection, the size of the company, the industry classification and the nature of ownership as shown in Table 8.

**Table 8:** Regression results of firm heterogeneity.

Variables	(1)	(2)	(3)	(4)	(5)	(6)	(7)	(8)
	Environmental investment level		Enterprise size		Industry classification		Nature of ownership	
	High environmental investment	Low investment in environmental protection	Large companies	Small and medium-sized enterprises	Manufacturing	Non-manufacturing	State-owned enterprises	Non-state owned enterprises
Dig	0.038***	0.026*	0.058***	0.027*	0.035***	0.071**	0.049***	0.034***
	(3.85)	(1.79)	(4.74)	(1.96)	(4.37)	(2.18)	(2.86)	(3.81)
Size	10.504***	9.945***			9.527***	16.325***	12.220***	9.986***
	(18.68)	(19.84)			(25.78)	(18.04)	(23.54)	(23.31)
Lev	0.263***	0.393***	0.281***	0.788***	0.414***	− 0.462**	0.0720	0.490***
	(3.52)	(5.06)	(3.23)	(10.36)	(8.49)	(− 2.39)	(0.75)	(8.45)
Age	− 0.105	0.0140	0.170	0.181	− 0.0780	0.567**	0.017	− 0.118
	(− 1.03)	(0.10)	(1.20)	(1.36)	(− 1.00)	(1.97)	(0.09)	(− 1.37)
Shrcr1	− 0.000	− 0.002	0.002*	− 0.005***	0.001	0.004	− 0.001	0.001
	(− 0.24)	(− 1.33)	(1.92)	(− 2.82)	(1.58)	(1.07)	(− 0.40)	(1.25)
Board	0.046	− 0.131	0.064	− 0.084	− 0.029	0.001	− 0.001	− 0.024
	(0.71)	(− 1.51)	(0.82)	(− 0.92)	(− 0.57)	(0.01)	(− 0.01)	(− 0.37)
Independent	− 0.309*	− 0.361	− 0.195	− 0.588**	− 0.271*	0.924*	− 0.058	− 0.297
	(− 1.66)	(− 1.40)	(− 0.94)	(− 2.13)	(− 1.87)	(1.81)	(− 0.24)	(− 1.57)
Roa	2.986***	1.817***	2.252***	2.437***	2.477***	1.915***	2.522***	2.446***
	(18.14)	(11.70)	(14.91)	(16.59)	(27.05)	(4.08)	(12.85)	(22.61)
Year	Control	Control	Control	Control	Control	Control	Control	Control
Firm	Control	Control	Control	Control	Control	Control	Control	Control
_cons	− 23.281***	− 21.361***	7.177***	7.966***	− 20.274***	− 43.039***	− 27.754***	− 21.584***
	(− 12.72)	(− 14.11)	(6.68)	(16.77)	(− 18.36)	(− 13.54)	(− 16.24)	(− 16.69)
N	1411	1339	1351	1399	2392	358	980	1770
R^2^	0.961	0.938	0.928	0.894	0.957	0.958	0.957	0.949

T-statistics are shown in parentheses. ⁎⁎⁎, ⁎⁎ and * indicate 1%, 5% and 10% respectively.

#### Heterogeneity in the level of corporate environmental investment

The application of digital technology can achieve rapid and optimal allocation and regeneration of resources, deepen the digital application of the manufacturing process, optimize the technical resources of enterprises for green innovation, empower green manufacturing, and achieve high-quality economic development. And the level of importance companies place on sustainability directly determines whether they implement green innovation. If companies invest in environmental protection with more capital, they can also use technology and other resources to carry out the corresponding technological innovation, and the digital transformation will have a greater incentive effect on companies' green innovation, the more productive they are. Conversely, companies that do not pay attention to the impact of environmental investments on corporate value will not significantly improve their level of green innovation, even if they undergo digital transformation. Therefore, the regression is grouped by the high level of corporate environmental investment. The regression results in columns (1)–(2) of Table 8 show that the improvement of digitalization level of heavy polluting enterprises with high level of environmental protection investment can more significantly motivate enterprises to engage in green technology innovation activities, while enterprises will seek the path to improve digitalization level to promote green production in heavy polluting enterprises, support innovative technology resources, and improve the level of green technology innovation in enterprises, thus increasing the productivity of heavy polluting enterprises.

#### Heterogeneity of firm size

Digital transformation may produce significant differences in total factor productivity for firms of different sizes, and the regression results in columns (3)–(4) of Table 8 show that both large firms and SMEs undergoing digital transformation can significantly improve their total factor productivity, but the improvement is more pronounced for large firms. This result shows that digital transformation has a scale effect, and the larger the company, the more "powerful" it is in terms of digital transformation. Possible reasons for this are that a digital transformation strategy for enterprises requires new types of technological investment, sufficient innovation capacity, strong risk resistance and a large number of complex talents, and that fierce market competition will stimulate large enterprises to make changes and optimize themselves by advancing digitalization. Compared with large enterprises, SMEs lack the ability to innovate, face numerous difficulties in terms of asset ownership, talent and policy support, and lack core competitiveness. As a result, larger companies are more capable of digital transformation and have a greater impact on business performance.

#### Heterogeneity of industry classification

Digital transformation may be influenced by the overall level of development of the different industries in which companies are located, and therefore the economic consequences of digital transformation show differences in different industries. According to the source of the sample of heavy polluters selected in this paper, all samples were divided into two groups: manufacturing and non-manufacturing. The regression results in columns (5)–(6) of Table 8 show that the digital transformation of non-manufacturing and manufacturing firms has a positive impact on the total factor productivity of firms. It can be found that the digitalization of non-manufacturing enterprises has a stronger effect on total factor productivity. Possible causes: On the one hand, non-manufacturing enterprises are more autonomous in their operations, leading them to take more initiative in coordinating with multiple external actors and seeking more cooperation, so as to obtain more relevant and complementary resources and effectively integrate internal and external resources; on the other hand, the fierce competition forces non-manufacturing enterprises to carry out digital transformation in all aspects, angles and chains, catalyzing the multiplier effect of digital transformation on productivity. This also fully illustrates the need to strengthen the deep integration of digital technology and manufacturing, and accelerate the digital transformation of manufacturing.

#### Heterogeneity in the nature of business ownership

Digital transformation may have heterogeneous effects on the total factor productivity of firms with different ownership, and this paper divides all samples into two groups: state-owned enterprises and non-state-owned enterprises. Columns 7 and 8 of Table 8 present the results of the analysis of firm ownership heterogeneity. The results show that digitization has a significant impact on total factor productivity for both state-owned and non-state-owned firms, but state-owned firms exhibit a more pronounced significance. Possible reasons are that, on the one hand, given the unique nature of their ownership, state-owned enterprises (SOEs) are better equipped to implement policies, and SOEs carry out a range of activities to digitally transform themselves in order to advance the goal of supply-side structural reform, and therefore, their digital transformation is faster and more advanced than that of non-SOEs. On the other hand, compared with non-SOEs, SOEs have sufficient stocks of resources, research technologies, talents, scale, and policies^2,31^ to create sufficient conditions for digital transformation and to respond quickly to the demand for digital transformation resources, allowing SOEs to play a more important role in promoting total factor productivity improvement.

### Mechanism analysis of the influence of digital transformation on the total factor productivity of enterprises

This paper presents an empirical analysis of the impact mechanism using a mediating effects model. The logarithm of the number of green patents independently filed by firms (GI) is used as a mediating variable to test the mechanism path of green technology innovation drive; the total CSR evaluation score (CSR) in Hexun.com was used as a proxy variable to measure CSR fulfillment to test the mechanism path of CSR. The WEISS model was used to measure the degree of cost stickiness of firms as a proxy variable to test the mechanism path of cost stickiness. There are many methods on testing mediating effects, and this paper uses a three-step approach to construct a mediating effects model: In the first step, the impact of digital transformation of enterprises on total factor productivity of heavy polluters is examined; The second step is to test whether digital transformation can significantly improve green technology innovation and corporate social responsibility and curb corporate cost stickiness; The impact of digital transformation, green technology innovation, corporate social responsibility and cost stickiness variables on a firm's total factor productivity when placed simultaneously in the regression equation is examined. The specific model is constructed as follows:

2 $$tfp_{i,t} = \alpha_{0} + \alpha_{1} Dig_{i,t} + \sum {\alpha_{n} } controls_{i,t} + F{\text{irm}}_{i} + year_{t} + \varepsilon_{i,t}$$

3 $$INTER_{i,t} = \beta_{0} + \beta_{1} Dig_{i,t} + \sum {\beta_{n} } controls_{i,t} + Firm_{i} + year_{t} + \varepsilon_{i,t}$$

4 $$tfp_{i,t} = \gamma_{0} + \gamma_{1} INTER_{i,t} + \gamma_{2} Dig_{i,t} + \sum {\gamma_{n} } controls_{i,t} + F{\text{irm}}_{i} + year_{t} + \varepsilon_{i,t}$$

where INTER denotes the mediating variable.

According to the regression results reported in column (2) of Table 9, it can be seen that the coefficient of the effect of digital transformation of enterprises (Dig) on the level of green technological innovation (GI) is 0.02839 which is significantly positive at the 5% level. suggest that with the further implementation of the digital transformation strategy of enterprises, the green technology innovation capability also increases significantly, as shown by the results of the empirical regression of the mediating effect of green technology innovation in column (3): the coefficient of the number of green patents is significantly positive and the reported coefficient of the impact of digital transformation on total factor productivity becomes 0.03727, with a decrease in the impact coefficient from the coefficient of 0.03842 in column (1) of the model estimation results, and the significance still passes at the 1% level. Suggesting that the level of green technology innovation partially mediates the effect between digital transformation and total factor productivity of heavily polluting firms. That is, digital transformation improves total factor productivity of heavy polluters by enhancing green technology innovation, and hypothesis 2 is verified. It can be seen that, as mentioned in the theoretical analysis, as the government pays more and more attention to eliminate the negative impact of heavy polluting enterprises on the environment, heavy polluting enterprises also pay more and more attention to technological innovation in green environment and invest more and more in green technological innovation, which is an effective way to achieve a win–win situation for both economic and environmental benefits of enterprises through digitalization. On the one hand, according to the theory of resource-based view, enterprises can promote the pooling and integration of heterogeneous knowledge and resources through digital transformation, among the heterogeneous resources obtained by heavy polluting enterprises, especially green technology resources, which expands the application boundary of enterprises to improve the level of green technology innovation, and data would have been efficient, immediate and dynamic, the application of digital technology in heavy pollution enterprises to promote the ability of enterprises to adapt to changes in the external environment, improve the efficiency of resource allocation and real-time monitoring of the ecological environment, so as to enhance the comprehensive ability of the level of green technology innovation; on the other hand, the improvement of enterprise green technology innovation level can make enterprises change the traditional simple and crude high pollution and high emission economic model, and form a closed-loop green economy of resources, And it is easier to obtain external financing and internal financial support, and the economic benefits generated can compensate for the input costs, improve the competitive advantage in the market, achieve win–win economic-social-environmental benefits, and thus improve the total factor productivity of the enterprise.

**Table 9:** Regression results of intermediate effects.

Variables	(1)	(2)	(3)	(4)	(5)	(6)	(7)
	tfp	GI	tfp	CSR	tfp	JSTICKY	tfp
Dig	0.03842***	0.02839**	0.03727***	1.2161**	0.03736***	− 0.05297*	0.03779***
	(3.7616)	(1.9662)	(3.6668)	(2.3659)	(3.6472)	(− 1.6867)	(3.7104)
Size	10.918***	− 0.8966	10.955***	54.198***	10.871***	− 0.7573	10.909***
	(11.9706)	(− 1.3836)	(12.0619)	(2.5872)	(11.9128)	(− 0.5066)	(11.9897)
Lev	0.3417***	− 0.01468	0.3423***	− 4.3881	0.3455***	− 0.02395	0.3414***
	(3.8974)	(− 0.1556)	(3.9145)	(− 1.2991)	(3.9335)	(− 0.0947)	(3.8908)
Age	− 0.1319	0.2500*	− 0.1420	0.6681	− 0.1325	− 0.01939	− 0.1322
	(− 1.0366)	(1.7616)	(− 1.1150)	(0.1281)	(− 1.0307)	(− 0.0618)	(− 1.0341)
Shrcr1	0.0004051	− 0.001094	0.0004493	0.02957	0.0003793	− 0.001993	0.0003813
	(0.2667)	(− 0.8172)	(0.2972)	(0.4919)	(0.2499)	(− 0.6068)	(0.2533)
Board	− 0.01624	− 0.03642	− 0.01477	− 4.1348	− 0.01263	0.4290**	− 0.01113
	(− 0.2676)	(− 0.3623)	(− 0.2466)	(− 1.1012)	(− 0.2093)	(2.4531)	(− 0.1837)
Independent	− 0.1873	0.09033	− 0.1910	− 16.409	− 0.1730	0.3509	− 0.1831
	(− 1.0568)	(0.3136)	(− 1.0983)	(− 1.6401)	(− 0.9763)	(0.6304)	(− 1.0379)
Roa	2.5193***	0.2064	2.5110***	78.220***	2.4510***	− 1.8530***	2.4972***
	(14.1142)	(1.3734)	(14.0796)	(11.9084)	(13.0949)	(− 3.2841)	(14.0385)
GI			0.04040***				
			(3.1696)				
CSR					0.0008728*		
					(1.6536)		
JSTICKY							− 0.01193*
							(− 1.8818)
Year	Control	Control	Control	Control	Control	Control	Control
Firm	Control	Control	Control	Control	Control	Control	Control
_cons	− 24.624***	2.3450	− 24.719***	− 130.17*	− 24.511***	2.1652	− 24.599***
	(− 9.0508)	(1.1502)	(− 9.1304)	(− 1.9441)	(− 9.0131)	(0.4879)	(− 9.0709)
N	2750	2750	2750	2750	2750	2750	2750
R^2^	0.6357	0.02147	0.6374	0.2621	0.6364	0.01778	0.6366

T-statistics are shown in parentheses. ***, ** and * indicate 1%, 5% and 10% respectively.

According to the regression results reported in column (4) of Table 9, it can be seen that the coefficient of impact of corporate digital transformation (Dig) on corporate social responsibility (CSR) is 1.2161, which passes the significance test at the 5% level. Further, after the inclusion of the mediating variable CSR, column (5) of Table 9 shows that the coefficient of the impact of digital transformation on the total factor productivity of firms becomes 0.03736, the impact coefficient decreases compared to the coefficient of 0.03842 in column (1) of the model estimation results, and the significance still passes at the 1% level. It shows that CSR has a partially mediating effect between digital transformation and the full range of corporate factors. It is shown that with the digital transformation of enterprises can enhance the willingness and ability of corporate social responsibility, make enterprises take more social responsibility and steadily improve the total factor productivity of enterprises, and hypothesis 3 is verified. It can be seen that the application of digital technology enhances the awareness and ability of enterprises to assume social responsibility, as described in the theoretical analysis, and that CSR is an internal driver of total factor productivity of enterprises through digitalization. According to the resource dependency view and stakeholder theory, enterprises taking more social responsibility can obtain more resources from outside and gain support from various stakeholders, enabling them to carry out a series of green innovation activities, promote pollution reduction and production reform, promote sustainable development, and boost enterprise total factor productivity.

According to the regression results reported in column (6) of Table 9, it can be seen that the coefficient of impact of digital transformation of firms (Dig) on the cost stickiness of firms (JSTICKY) is -0.05297, which passes the significance test at the 10% level. Column (7) shows that cost stickiness and firm total factor productivity are significantly negative at the 1% level, indicating that high cost stickiness inhibits the development of a firm's profit margin and makes it difficult to enhance firm total factor productivity. After incorporating the mediating variable cost stickiness, column (7) of Table 9 shows that the coefficient of the impact of digital transformation on the total factor productivity of firms becomes 0.03779, the impact coefficient decreases from the coefficient of 0.03842 in column (1) of the model estimation results, the sign does not change and the significance still passes at the 1% level. It shows that cost stickiness has a partially mediating effect between digital transformation and the full factor of the enterprise. The above results generally validate that the mechanism of cost stickiness holds true, and that digital transformation can improve the efficiency of capital turnover and improve problems such as idle resources and inventory backlogs, which can curb cost stickiness and thus increase the total factor productivity of a company. Hypothesis H4 is validated.

## Discussion

In the digital economy, companies have introduced a new generation of digital technologies for digital transformation. In this paper, we take the heavily polluting listed A-share companies in Shanghai and Shenzhen from 2010 to 2020 as our research target, and in the following we will discuss our main findings.

First: This study investigates the impact of digital transformation of heavy polluters on the total factor productivity of firms, using textual analysis and measuring the level of digitalization by digital investment, respectively. After a series of empirical regressions, the impact of digital transformation on the total factor productivity of heavy polluters is also found to be significant. Previous studies have investigated the impact of digital transformation on the total factor productivity of enterprises for all listed companies, but this paper is limited to heavily polluting enterprises and confirms that there is a positive impact of the digital transformation of heavy pollution on the total factor productivity of enterprises. This shows that digital transformation has become a necessary path for high-quality development of enterprises. As the proposal of China's carbon peak and carbon neutral targets has aroused concern in the domestic and international community, Chinese heavy polluters must pay attention to the important role of digital transformation, applying digital technologies such as big data, cloud computing, blockchain and the Internet of Things to shift towards smart manufacturing, while applying internet business thinking in their operations, building efficient information systems and helping to achieve the goal of carbon peaking and carbon neutrality with the help of digital technologies and their own planning.

Secondly: the implementation of digital transformation enables companies to achieve improvements in total factor productivity by increasing their level of green technology innovation and their willingness to improve their corporate social responsibility and reduce their cost stickiness. The heavily polluting enterprises support the innovation and development of green technology through data elements and digital technology, improve the sustainable development ability of enterprises with greener and lower-carbon emissions and outputs, and improve the total factor productivity of enterprises. Externally, by enhancing the willingness and ability of corporate social responsibility, enterprises can better undertake corporate social responsibility, which can promote the full disclosure of corporate information and effectively realize the coordination of stakeholders, but the technology and reputation of enterprises are also improved accordingly, resulting in social spillover effects. In cost management, companies are driving total factor productivity by improving digital transformation to improve adjustment costs, agency costs and managers' optimistic expectations and curb cost stickiness. At present, China's economy has shifted from the stage of high speed growth to the stage of high quality development, and the important micro foundation for promoting high quality economic development is enterprises. As for the heavily polluting enterprises, in the process of providing total factor productivity of enterprises, we should focus on innovation leadership and drive sustainable development of enterprises through green technology innovation. At the same time, the digital strategy should be aligned with the social responsibility strategy to better exploit the network effect of value sharing through more active social responsibility, and also to optimise resource allocation and improve cost control through digital transformation, thereby continuously improving the overall value of the enterprise.

Finally, we found that the impact of implementing digital transformation in companies is heterogeneous across companies. First, for state-owned enterprises, the impact of digital transformation on the total factor productivity of enterprises is more significant. State-owned enterprises themselves have the advantage of being embedded in a good national credibility chain, are more proactive in implementing relevant policies, are more likely to gather capital, information and talent, and will accordingly be susceptible to the attention of the state. Secondly, the digital transformation of non-manufacturing companies has a stronger effect on the total factor productivity of companies because non-manufacturing companies operate with more flexibility and are more likely to focus on technology and data elements, while manufacturing companies are more concerned with factors such as human and capital. Thirdly, the impact of digitisation on total factor productivity of enterprises is more significant for large-scale enterprises, as large-scale enterprises have higher market credibility and capital absorption than small-scale enterprises, and thus the impact of digital transformation on total factor productivity of large-scale enterprises is obvious. Fourth, in the study of heavy polluters, this paper finds that the impact of digital transformation on total factor productivity is more significant in firms with high levels of environmental investment. With the increasing investment in environmental protection, the waste treatment capacity of enterprises will be enhanced and the production process will be optimised, which will help to improve the efficiency of resource utilisation and enhance the competitiveness of enterprises, thus leading to a further increase in total factor productivity. Driven by China's current "double carbon" target, enterprises need to actively take responsibility for environmental protection, continuously increase investment in environmental protection and accelerate the replacement of environmental protection equipment.

## Conclusion and insights

### Research conclusions

This paper discusses the impact of digital transformation on the total factor productivity of heavy polluters and its mechanism of action through theoretical analysis and empirical testing. Based on the discussion in Part 5, the following research conclusions were drawn. ① Digital transformation of heavily polluting enterprises can improve total factor productivity of enterprises overall. ② As for the mechanism of action, the mechanism of the impact of digital transformation on the total factor productivity of enterprises is mediated through green technological innovation, corporate social responsibility and cost stickiness. ③ The impact of digital transformation on a firm's total factor productivity shows a significant time lag effect, which indicates that the impact of digital transformation initiatives implemented by firms in the current period on their total factor productivity is lagged; ④ Heterogeneity analysis shows that the effect of digital transformation on the total factor productivity of enterprises is more pronounced for state-owned enterprises, non-manufacturing enterprises, heavy polluters with high level of environmental investment and large scale. The study, however, also has some limitations. First, there is a single variable measure of digital transformation. This paper uses textual analysis and digital investment to measure the degree of digital transformation of enterprises, but has not yet achieved a detailed portrayal of the digital evolution of many different aspects of production, R&D and sales. Future research could try to establish a scientific system of indicators to measure the digital transformation of enterprises using ICT technologies and ICT services, etc. Secondly, the paper is not comprehensive and well developed in sorting out the theoretical mechanisms of the impact of digital transformation on the total factor productivity of enterprises. In the future, the potential mechanisms through which digital transformation in various segments affects the total factor productivity of enterprises can be dissected from different perspectives, such as resource allocation, technological progress, operational efficiency and transaction costs; Furthermore, in response to the call for environmental protection, it may be interesting to examine the further relationship between digitalisation and green total factor productivity (GTFP). Finally, in the empirical analysis, only the fixed effects model and the mediating effects model are used for the regression analysis, which may lead to a lack of depth in the research of this paper. Influenced by the policy, some companies have proposed digital transformation development strategies. As policies are often exogenous to the decisions of micro-subjects, the pilot policies of the 12th Five-Year Plan for the Development of National Strategic Emerging Industries and the Implementation Plan for the Broadband China Strategy can be used as quasi-natural experiments in future research. The use of double difference methods to estimate the impact of digital transformation on a firm's total factor productivity also goes some way to avoiding endogeneity problems.

### Countermeasures and suggestions

The following insights can be drawn from this study:

1. The government and relevant departments should introduce a special policy system to implement the "Broadband China" strategy, broaden the coverage of digital infrastructure, and create a favourable external environment for the digital transformation of heavy polluters. On the one hand, government authorities should encourage and promote the application of digital technology in the production and operation of enterprises and other aspects, provide corresponding services for the digital transformation of enterprises, and assist in building a digital ecosystem with upstream and downstream collaboration across the industrial chain. and introduce support and subsidy policies for enterprises in the process of digital transformation to alleviate the difficulties in financing green technology innovation and the shortage of talents; on the other hand, in the process of helping enterprises' digital transformation, the digital transformation of non-state enterprises and manufacturing enterprises has no significant effect on total productivity improvement through heterogeneity analysis in this paper. Therefore, government departments should promote digital development in this field, "fill the short board", while "forging the long board" for key industrial areas, to achieve a comprehensive, balanced, high-quality digital transformation; Finally, in the digital ecological development environment, enterprises should pay more attention to the formation of digital protection mechanisms, and the government should further improve the construction of digital security systems to promote the sharing of big data resources under the premise of safeguarding information security.
2. Emphasis on the role of the application of digital technology to enhance the level of heavy pollution green technology innovation. The mechanism analysis shows that green technology innovation as an influencing mechanism of digital transformation to drive total factor productivity of enterprises. Therefore, on the one hand, enterprises should recognize the important role of green technology innovation in digitization for high-quality development of enterprises, stimulating their R&D and production capacity in green technology innovation, and actively applying digital technologies in manufacturing, energy management, supply chain, and innovation processes. Introduce digital simulation, data management, big data analysis and other technologies in green technology innovation and energy saving, consumption reduction and emission reduction to achieve intelligent collaboration. Establish a** s**ustainable development R&D and innovation system based on digital technology, and empower the application of intelligent production and manufacturing on green innovation in heavy polluters with digital technology. Actively use existing digital technologies to optimise traditional production processes, promote business process re-engineering in enterprises, build smart factories and smart workshops within enterprises, realise unmanned management and intelligent production, accelerate the quality and operational efficiency of products produced by enterprises, and enterprises should accelerate the construction of industrial data chains for the industrial chain and product life cycle in the production process, and further improve the digitisation rate of key processes to realise The empowering and efficiency-enhancing effect of digitisation on the sustainable development of heavily polluting enterprises to promote green modernisation and productivity improvement. On the other hand, relevant departments should create a good market environment, strengthen policy support and institutional protection for green development, and ensure fair competition for enterprises in green technology innovation;
3. Heavy polluters should take the initiative to fulfill their social responsibility to improve the total factor productivity of enterprises. On the one hand, giving full play to corporate responsibility for environmental governance, improving their own environmental management and adjusting their business strategies to actively cooperate with the implementation of different types of environmental policies. We should not only focus on economic performance in terms of financial responsibility to maximize the benefits for shareholders, suppliers and employees, but also actively fulfill our responsibilities to government, society and the environment in order to gain a good market reputation and enhance our overall competitiveness. On the other hand, environmental protection departments should strengthen supervision, pay timely attention to the social responsibility performance of heavy polluters, and improve CSR assessment standards and systems for heavy polluters to make the CSR assessment process comprehensive, transparent and standardized. At the same time to achieve both reward and punishment, the fulfillment of social responsibility as an assessment index into the regular assessment of enterprises, so that enterprises in the pursuit of economic benefits at the same time pay more attention to social and environmental benefits.
4. The following recommendations are made to further improve the digitalisation of enterprises and to reduce the negative impact of cost stickiness on the total factor productivity of enterprises. Firstly, cost stickiness is prevalent in enterprises, inhibiting total factor productivity and requiring sound internal control systems to improve cost control efficiency and reduce waste of resources. Secondly, we actively plan for digital transformation, promote digital transformation in all aspects of product, production and management, and optimise the allocation of enterprise resources. Cost stickiness reveals, to a certain extent, the redundancy of enterprise resources, and the promotion of enterprise technology and management innovation is conducive to improving the efficiency of enterprise resource allocation. Finally, government departments should create a development environment that is conducive to the digital transformation of enterprises, optimize the governance system, strengthen the supervision of managers' behaviour and prevent them from making excessive investments with optimistic expectations. Finally, government departments should create a development environment that is conducive to the digital transformation of enterprises, optimize the governance system, strengthen the supervision of managers' behaviour and prevent them from making excessive investments with optimistic expectations. Special support policies and incentive mechanisms are also in place to address the agency issues between the company's shareholders and management.

## Data Availability

The data presented in this study are openly available in the China Research Data Service Platform (CNRDS), the China Stock Market Accounting Research Database (CSMAR), ChinaHub official website (https://www.hexun.com/) and annual Report Manual Collation. The data used to support the findings of this study are available from the corresponding author.
